# Anti-Cancer Immunotherapies Targeting Telomerase

**DOI:** 10.3390/cancers12082260

**Published:** 2020-08-12

**Authors:** Simone Negrini, Raffaele De Palma, Gilberto Filaci

**Affiliations:** 1 Internal Medicine, Clinical Immunology and Translational Medicine Unit, IRCCS Ospedale Policlinico San Martino, 16132 Genoa, Italy; raffaele.depalma@unige.it; 2 Centre of Excellence for Biomedical Research and Department of Internal Medicine, University of Genoa, 16132 Genoa, Italy; gfilaci@unige.it; 3 Biotherapy Unit, IRCCS Ospedale Policlinico San Martino, 16132 Genoa, Italy

**Keywords:** telomerase, hTERT, immunotherapy, vaccine, cancer, clinical trials

## Abstract

Telomerase is a reverse transcriptase that maintains telomeres length, compensating for the attrition of chromosomal ends that occurs during each replication cycle. Telomerase is expressed in germ cells and stem cells, whereas it is virtually undetectable in adult somatic cells. On the other hand, telomerase is broadly expressed in the majority of human tumors playing a crucial role in the replicative behavior and immortality of cancer cells. Several studies have demonstrated that telomerase-derived peptides are able to bind to HLA (human leukocyte antigen) class I and class II molecules and effectively activate both CD8^+^ and CD4^+^ T cells subsets. Due to its broad and selective expression in cancer cells and its significant immunogenicity, telomerase is considered an ideal universal tumor-associated antigen, and consequently, a very attractive target for anti-cancer immunotherapy. To date, different telomerase targeting immunotherapies have been studied in pre-clinical and clinical settings, these approaches include peptide vaccination and cell-based vaccination. The objective of this review paper is to discuss the role of human telomerase in cancer immunotherapy analyzing recent developments and future perspectives in this field.

## 1. Introduction

Telomeres are specialized nucleoprotein structures that are located at the termini of linear chromosomes, and composed of repetitive nucleotide sequences (5′-TTAGGG-3′) and associated with a complex of six protein components (TRF1, TRF2, Rap1, TIN2, TPP1, and POT1), named shelterin. Telomeres protect the ends of chromosomes from being misrecognized as DNA breaks thus preventing dangerous consequences of unwanted DNA damage responses, the so-called “end-protection problem” [1]. In normal eukaryotic cells, telomeres shorten with each cell division because DNA polymerase is unable to replicate the 3′ end of the DNA lagging strand, a situation known as the “end-replication problem” [2]. In particular conditions, this phenomenon is counterbalanced by telomerase, a ribonucleoprotein complex comprising the telomerase reverse transcriptase (TERT), the telomerase RNA component (TERC) and other associated proteins. TERT adds short repetitive DNA sequences to the telomeric regions of chromosomes using its own RNA as template, thus preventing chromosome shortening [3].

Telomeres shortening is strictly related to cellular senescence, a physiologic process that occurs in the majority of human cells, since they do not express telomerase. In humans, telomerase expression is limited to stem cells or germline cells, but it is virtually absent in normal adult tissues. On the other hand, telomerase activity can be detected in 85–90% of human cancers at every stage of tumor development, from cancer stem cells to metastatic cancer cells, and is thought to play a central role in the replicative behavior and immortality of cancer cells [4,5,6]. This notion is supported by experiments on the genetic modifications required to convert normal human cells to a fully tumorigenic state. Indeed, transduction of normal cells by retroviruses encoding two oncogenes (the simian virus 40 large T oncoprotein and an oncogenic RAS protein) and human TERT (hTERT) is sufficient to convert normal human epithelial and fibroblast cells into fully tumorigenic cells, whereas cells transduced without hTERT did not produce tumors [7]. In addition, TERT inhibition in cell culture led to telomere shortening, as well as cellular apoptosis and inhibition of cancer cell growth, thus providing additional evidence that telomerase is fundamental for cancer cell immortalization and tumor progression [8,9,10]. Besides the main canonical function related to telomere maintenance, telomerase displays different non-canonical activities including regulation of gene transcription, resistance to anti-growth signals, cell proliferation, β-catenin/MYC/NF-κB signaling, angiogenesis and epithelial-mesenchymal transitions (EMT), this latter being deeply implicated in migratory and invasive properties of cancer cells [11,12]. On the whole, this data indicates that telomerase, through its canonical and non-canonical functions, represents a central regulator of all of the hallmarks of cancer [12]. The crucial role of telomerase in tumor growth and development suggests that hTERT down-modulation as a means of immune escape may itself have deleterious effects on cancer cells. Recently, comprehensive whole-genome analysis has identified that the accumulation of point mutations within the promoter region of the hTERT gene is responsible for the increased TERT expression [13]. Only in a minority of cancer, telomere length maintenance is achieved by telomerase-independent mechanisms, referred to as “alternative lengthening of telomeres” (ALT) [14].

Due to its pivotal biological role in cancer, different strategies, other than immunotherapy, have been explored for targeting telomerase as anti-cancer therapy. These approaches include: G-quadruplex stabilizers (e.g., RHPS4, Telomestatin, TMPyP4, CX-3543/quarfloxacin), antisense oligonucleotides (e.g., Imetelstat /GRN163L), small-molecule inhibitors (e.g., BIBR1532) and oncolytic viruses (e.g., Telomelysin) [15,16,17,18].

The basic principle of cancer immunotherapy is based on the manipulation of the host immune system in order to attack the cancer cells. Over the past decades, scientific and technological advances in immuno-oncology have contributed to its role as one of the most promising approaches for the treatment of cancer thus making immunotherapy the fourth pillar of cancer care, complementing surgery, chemotherapy and radiotherapy. Cancer immunotherapy strategies (alone or in combination) includes: (i) Use of cytokines to increase the immune response; (ii) vaccine-based strategies that, through the administration of antigens or antigen-presenting cells (APCs), aim to indirectly amplify the effector component of immunity; (iii) delivery of effector cells, including “engineered” cells (e.g., chimeric antigen receptors-modified T cells), to directly expand the effector population; and (iv) manipulation of “co-stimulatory—inhibitory checkpoints” to shift the immune response toward activation [19].

In the context of vaccine-based strategies, the main challenge is represented by the identification of an appropriate tumor-associated antigen (TAA). An ideal TAA should display a selective expression in the majority of cancers, and in all phases of tumor progression, in order to be employed in a large number of patients without risk of autoimmune reactions and should have the capacity to elicit strong immune responses ideally involving both CD4^+^ and CD8^+^ T lymphocytes [20]. Due to its broad and selective expression in cancers cells, telomerase has been immediately considered an attractive and almost universal therapeutic target for anti-cancer immunotherapy, so that an important aspect of being confirmed was the capacity of hTERT-derived peptides to elicit an effective immune response.

The first identified hTERT immunogenic peptide, p540 (ILAKFLHWL), is restricted to the MHC (major histocompatibility complex)class I allele HLA-A*0201 (HLA-A2), the most prevalent HLA allele found among nearly 50% of Caucasians, Asians, and Hispanics and 33% of African-Americans. Investigators observed that in vitro immunization with p540 efficiently generated hTERT-specific cytotoxic T lymphocytes (CTLs) capable of killing different HLA-A2^+^ hTERT-expressing cancer cell lines and primary tumors cells [21,22]. In the following years, additional hTERT peptides processed and presented by cancer cells expressing different HLA-I allelic variants have been reported in the literature [23,24,25,26]. In addition to MHC I-restricted peptides, different peptides capable of binding class II molecules have been characterized, thus indicating that hTERT can also be efficiently presented to CD4^+^ T helper lymphocytes [27,28,29]. Many studies confirmed that healthy individuals and patients with cancer have circulating hTERT antigen-specific CTLs that can mediate an efficient lysis of tumor cells of multiple histological types [21,22,23,25,30,31,32,33]. Finally, pre-clinical data confirmed the possibility to expand the pool of hTERT peptide-specific CTLs in vivo through different vaccination approaches [21,23,31,34,35,36,37].

Telomerase is not expressed in normal adult somatic cells, but it can be detected in germline cells, stem cells and other cell types (e.g., B and T lymphocytes) [38,39,40,41]. This notion raised concern about the risk of autoimmune reactions induced by hTERT-targeting immunotherapies. However, different pre-clinical experimental data demonstrated that vaccinations targeting hTERT are safe, with no risk of autoimmune adverse events [21,22,34,42,43]. One explanation for this finding is that the level of hTERT expression in normal cells is below the threshold level found in malignant cells that are recognized and lysed by hTERT-specific CTLs.

All these encouraging preliminary data, paved the way for the development of clinical protocols using telomerase as an immunizing agent. In this review, we will discuss the current status of telomerase-targeting cancer therapeutics in clinical trials (Table 1).

## 2. Peptide Vaccines Targeting hTERT

In order to trigger adaptive immunity, antigens have to be degraded (processed) into small peptides and presented to immune cells in the context of major histocompatibility complex (MHC) molecules. MHC class I (MHC-I) molecules are expressed by virtually all nucleated cells and present peptides derived from intracellularly expressed proteins to cytotoxic CD8^+^ T cells. MHC class II (MHC-II) proteins are typically expressed by professional APCs, such as dendritic cells (DCs), and serve to present internalized exogenous protein to CD4^+^ T helper lymphocytes. DCs, through a process called cross-presentation, may also present exogenous antigens within MHC-I molecules, thus allowing the activation of cytotoxic CD8^+^ T cells. The first reports focusing on hTERT immunogenicity explored the capacity of hTERT-derived peptides to bind MHC-I molecules and activate CD8^+^ CTLs. To date, over 30 hTERT synthetic peptides have been described, and part of them have been employed in clinical trials targeting human telomerase [20]. The majority of these peptides are restricted to MHC-I molecule (thus, they are able to trigger CTLs), and some others can bind MHC-II molecules and can be exploited to induce Th cell responses.

### 2.1. GV1001

The GV1001 vaccine is based on a peptide sequence composed of 16 amino acids (611–626: EARPALLTSRLRFIPK) derived from the hTERT active site [44]. GV1001 is a highly promiscuous peptide capable of binding to molecules encoded by multiple alleles of all three loci of HLA class II and can also be further processed into CTL epitopes [45]. Therefore, differently from other single-peptide vaccines [46,47,48], GV1001 can be administered without the need of HLA-typing of patients and can induce an efficient hTERT-specific T cell response in vivo, involving both T helper and CTL responses [45,49]. In addition, the demonstration of T cell responses against several GV1001 unrelated epitopes suggests an “epitope spreading effect” induced by vaccination [45]. GV1001 vaccine is employed in association with granulocyte-monocyte colony stimulating factor (GM-CSF). GM-CSF has been demonstrated to mediate the recruitment and maturation of DC, as well as activation of macrophages, neutrophils and NK cells: As a consequence, it has been largely employed as immunostimulatory adjuvant in cancer immunotherapy [50]. Besides its immunogenic effects, GV1001 is capable to penetrates within tumor cells, where it can down-regulate heat shock protein (HSP) 90, HSP70, hypoxia-inducible factor (HIF)-1 and vascular endothelial growth factor (VEGF), thus increasing its anti-tumor effect [51,52,53].

In a phase I/II study, 48 patients with advanced pancreatic cancer received GV1001 at three dose levels in combination with GM-CSF. Immune responses were observed in 63% of the evaluable patients, with the highest ratio in the intermediate dose group (75%). Median survival for the intermediate dose group was significantly longer as compared to other groups, suggesting that the induction of an immune response is correlated with prolonged survival [54].

In the phase I/II CTN-2000 trial, Brunsvig and colleagues [49] treated 26 patients affected by non-small cell lung cancer (NSCLC) with GV1001 in combination with p540 hTERT peptide and GM-CSF. The investigators observed response to p540 in only 2 out of 24 patients, probably because patients were not pre-selected on HLA typing, but 11 out of 24 subjects responded against GV1001. In a long-term follow-up of the CTN-2000 cohort, 54% of the evaluable subjects developed a GV1001 response. The immune responders displayed increased survival compared with non-responders. Follow-up of four long-time survivors showed that they all developed stable GV1001-specific T cell memory responses along with pro-inflammatory cytokine profiles [55]. In a successive phase II study conducted by the same group, the CTN-2006 trial, 23 inoperable NSCLC patients received radiotherapy and docetaxel, followed by GV1001 vaccination. A GV1001-specific immune response was observed in 16/20 evaluable patients, and immune responders achieved a median progression-free survival (PFS) of 371 days, compared with 182 days for non-responders [55].

Hungher and co-workers treated a cohort of melanoma patients with GV1001 and p540 peptides in combination with GM-CSF or with GV1001 associated with tuberculin PPD23 as an alternative vaccine adjuvant. Interestingly, peptide-specific immune responses were detected in 7 of 10 patients of the first group, whereas no measurable immune responses were observed in the second group. To explain this result, the authors suggested that the immune response against mycobacterial peptides is dominant, resulting in a suppression of the immune response against the hTERT peptide [56].

Another trial on melanoma, explored the combinations of GV1001 with the alkylating agent temozolomide. The treatment induced an immune response in 18/23 evaluated subjects, five partial tumor regression, six stable disease, and one complete remission, thus supporting the idea of combining cancer vaccination with chemotherapy [57].

Staff et al. [58] studied GV1001 vaccination together with gemcitabine in advanced pancreatic cancer patients. Immune responses were observed in 8/12 patients when gemcitabine was administered concurrently with immunotherapy and in 2/5 patients when chemotherapy was added at disease progression but, on the whole, the immune responses were weak and transient. In addition, none of the 17 evaluable patients achieved a partial or complete response. Similarly, GV1001 vaccination for cutaneous T cell lymphoma (CTCL) failed to induce any objective clinical response in treated patients suggesting that the GV1001 vaccination is not effective in CTCL patients [59].

The effect of immunomodulatory Treg targeting chemotherapy (low-dose cyclophosphamide) in combination with GV1001 vaccination has been explored in patients with advanced HCC. However, no GV1001 specific immune responses were detected after vaccination, and none of the patients achieved a complete or partial response to treatment although 17/37 patients demonstrated a stable disease six months after initiation of treatment [60].

Pre-clinical data on pancreatic ductal adenocarcinoma, indicated that GV1001 associated with gemcitabine is capable of inducing significant loss of fibrosis in tumor tissue and tumor cell death [61]. However, a large phase III trial on metastatic pancreatic cancer patients showed that addition of GV1001 to chemotherapy (gemcitabine and capecitabine) did not translate into any significant clinical benefits for the vaccinated patients. Indeed, comparing sequential or concurrent chemoimmunotherapy with the chemotherapy alone, it emerged that GV1001 failed to improve median time to progression (4.5 months and 6.6 months, respectively, versus 6.4 months), overall survival (6.9 months and 8.4 months, respectively, versus 7.9 months) and objective response rates (9% and 16%, respectively, versus 18%). Twelve of 32 patients (38%) on sequential immunotherapy and 25 of 68 (37%) patients on concurrent chemoimmunotherapy had a complete immune response, but these responses were not associated to any clinical improvement [62].

### 2.2. GX301

GX301 vaccine is constituted by four promiscuous immunogenic hTERT peptides (peptide 540–548, peptide 611–626, peptide 672–686 and peptide 766–780) that can bind to both HLA class I and class II molecules. GX301 is administered with two adjuvants Montanide ISA-51 and Imiquimod. Montanide generates a water-in-oil emulsion that protects peptides from tissue proteases and facilitates peptides uptake by intradermal DCs. Additionally, it induces IFNγ release by innate immunity cells, increasing the expression of MHC molecules by tumor cells [63]. Imiquimod is a strong activator of the Toll-like receptor (TLR) 7 and 8, inducing activation and maturation of DCs [64,65]. Collectively, the two adjuvants exert complementary functions promoting simultaneous uptake and presentation of vaccine peptides. With respect to single peptide vaccinations, the multi-peptide composition of the GX301 vaccine offers substantial benefits in terms of immunogenicity, extended HLA-haplotype coverage and inductions of both CD4^+^ and CD8^+^ T cells responses. Accordingly, a study, aimed at investigating the immunogenicity of the telomerase peptides included in GX301 vaccine in the healthy population, showed that 100% of healthy subjects were able to mount an ex vivo effector immune response against at least one of the four peptides. Hence, this data demonstrated that immune tolerance does not significantly affect immune responses against telomerase, although it is an endogenous antigen [66].

Our group conducted a phase I clinical trial using a GX-301-based vaccination protocol in patients with stage IV prostate cancer or renal cancers. All treated patients exhibited a vaccine-specific immunologic response to at least one of the peptides. Disease stabilization occurred in four patients, and prolonged PFS and OS were observed in patients showing a full pattern of vaccine-specific immunologic responses [67]. A multicenter phase II trial (NCT02293707) is currently ongoing to confirm our preliminary observations on efficacy and safety of GX-301 and to determine the most effective administration protocol in patients with progressive, castration-resistant prostate cancer [68].

### 2.3. UV1

In the context of multi-peptide vaccination strategies, UV1 is a second-generation telomerase-based vaccine constituted by three hTERT-derived peptides (peptide 652–665, peptide 660–689 and peptide 691–705), which are the most frequently recognized by CD4^+^ T cells of long term cancer survivors, based on epitope spreading following vaccination with GV1001. UV1 has been recently tested in a phase I/II trial for patients with metastatic hormone-naïve prostate cancer in combination with radiotherapy and androgen deprivation treatment (ADT). An immune response was observed in 86% of the patients, 64% of the subjects achieved disease stabilization and 45% of the patients had no evidence of disease at the end of the trial [69]. Moreover, due to the study design, it was not clear whether the clinical benefits were attributable to the vaccine or, at least in part, to concomitant ADT and radiotherapy [70].

Different clinical trials are currently registered to evaluate the safety and efficacy of UV1 in association with single or multiple checkpoint blockade. Examples of checkpoint inhibitors associated to UV1 include: Ipilimumab (anti-CTLA-4) or pembrolizumab (anti-PD-1) in melanoma patients (NCT02275416 and NCT03538314, respectively), and ipilimumab in association with nivolumab (anti-PD-L1) in patients affected by mesothelioma (NCT04300244) or melanoma (NCT04382664).

### 2.4. Vx-001

Peptides derived from antigen processing can be broadly divided in two categories: Dominant and cryptic peptides, according to their affinity for the MHC, which in turn, influences the likelihood of each peptide to be efficiently presented. During thymic selection, developing T cells which recognize self-peptide/self-MHC complexes with high affinity are clonally deleted. Conversely, cryptic peptides are not efficiently presented, and consequently, T cells specific for cryptic self-peptides can escape thymic selection. As a result, cryptic peptides can be recognized by the immune system as non-self, since they escape thymic self-tolerance. On the other hand, cryptic peptides, because of their low MHC affinity, are very weakly immunogenic and cannot be employed in their native form to stimulate an efficient antitumor immune response. This problem can be circumvented by increasing MHC affinity of cryptic peptides via amino acids substitutions, thus making these “optimized cryptic peptides” highly immunogenic [71,72].

Vx-001 is the first antitumor vaccine based on optimized hTERT cryptic peptides, and is composed of two separate peptides: The native cryptic peptide ARG-Vx001 (p572) and its optimized variant TYR-Vx001 (p572Y) [73]. The two peptides are administered sequentially, in combination with Montanide. The vaccination protocol is based on first the administration of the optimized hTERT peptide (p572Y) followed by the native TERT 572 peptide. The rationale of this vaccination scheme relies on the hypothesis that subsequent vaccinations with the native TERT572 peptide may select and expand T cells with the highest avidity for the native peptide from the T cell pool previously primed by the optimized peptide [74].

In 2006, a pilot study assessing the safety and immunogenicity of the Vx-001 in various advanced tumors showed that this vaccine strategy was effective in eliciting specific CD8^+^ lymphocytes in 93% of the treated patients without any relevant toxicity [75].

In 2007, the same research group demonstrated that the vaccine was safe and capable of inducing early specific T cell responses in advanced NSCLC patients (76% and 91% of the patients after two and six vaccinations, respectively). In this study, the immunological response was significantly associated with longer time to progression and OS [76].

Similar positive data have been reported in another study on various types of chemo-resistant advanced solid tumors. In this trial, the authors demonstrated that Vx-001 was effective in inducing hTERT-reactive T cells in 51% of the patients after the 2nd vaccination and in 69% of the evaluable subjects after the completion of six vaccinations. Vaccination-induced immunological response was significantly correlated with increased survival in the subset of patients with disease progression at study entry [77].

In an expanded Phase II study, Vx-001 has been evaluated in patients with different advanced solid tumors. An hTERT-specific T cell immune response was observed in 55% and 70% of patients after the second and the sixth vaccinations, respectively. Immunologically responding patients had a better clinical outcome (PFS and OS) than non-responders [78].

Another Phase II study evaluated the clinical and immunologic response of patients affected by advanced NSCLC vaccinated with Vx-001. A specific immune response to the hTERT vaccine was observed in 66% of patients and OS, but not PFS, was significantly prolonged in immune-responders compared to those who failed to develop an immune response. Interestingly, the immune responses to Vx-001 and objective clinical responses were significantly higher in patients with a non-squamous histology, compared to those with squamous-cell carcinoma (SCC). This observation may be related to the occurrence in SCC of inactivating somatic mutations that may lead to alterations of HLA genes, potentially impairing antigen expression. Accordingly, a genotype-based selection of the patient candidates to immunotherapy may increase the probability of an effective anti-tumor response [79].

A very recent phase IIb trial exploring the clinical activity of Vx-001 as post-chemotherapy maintenance immunotherapy in advanced NSCLC failed to meet its primary endpoint, i.e., the increase in OS of vaccinated patients (median 11.3 for placebo and 14.3 months for Vx-001; *p* = 0.86). However, it is worth to note, that immune responder patients experienced a significant increase of OS (21.3 and 13.4 months, respectively; *p* = 0.004) and time to treatment failure (9.1 vs. 3.6 months, respectively; *p* = 0.0001) compared to non-responders. This study indicates that Vx-001 may confer clinical benefit in selected patients who can mount an adequate immune response upon vaccination, confirming the importance of defining the subgroup(s) of patients with the greater probability to respond to the vaccination in view of future clinical trials [80].

In a phase 1/2 two-arm trial, 54 patients with multiple myeloma received autologous stem cell transplantation followed by ex vivo activated autologous T cells. HLA-A2-positive patients (*n* = 28) also received pneumococcal conjugate vaccine immunizations, and a multi-peptide tumor antigen vaccine derived from the hTERT and survivin, whereas HLA-A*02-negative patients received the pneumococcal vaccine only. hTERT peptides employed for the immunization included hTERT p540 peptide and two optimized cryptic peptides (p572Y and p988Y). Immunological responses were observed in 10/28 of the subjects (36%) in the hTERT vaccine arm, but this cohort did not exhibit better clinical outcome [81]. This observation suggests that the presence of an immune response does not necessarily translate into an efficient anti-tumor response, but other factors (such as magnitude and quality of the immune response induced by vaccination) are important for achieving significant clinical effects.

Two additional multi-epitope vaccines based on optimized cryptic peptides have been recently patented: Vx-006, composed of three optimized cryptic peptides derived from three different TAA (MAGE, TERT, HER-2/neu), and Vbx-016 based on four optimized cryptic peptides derived from four tumor antigens (CEA, MAGE, TERT and HER-2/neu), but to date, no published results are available in the literature [82].

## 3. Dendritic Cell Vaccines

DCs are a heterogeneous population of immune cells characterized by phagocytic, antigen processing and antigen presentation capabilities. Through the activation of naive T cells, DCs initiate and orchestrate the adaptive immune response, and accordingly, play a pivotal role in anti-tumor immunology [83]. In preclinical models, immunization of mice with DCs transfected with TERT mRNA efficiently triggered CTLs competent to recognize and eliminate both murine and human tumor cells [34,35].

In a pioneering study published in 2004, Vonderheide and colleagues [84] demonstrated the induction of hTERT-specific lymphocytes in four out of seven patients with advanced breast or prostate carcinoma after vaccination with ex vivo generated autologous DC pulsed with the p540 hTERT peptide administered with keyhole limpet hemocyanin as an adjuvant. Interestingly, partial tumor regression observed in one patient was correlated with the induction of tumor infiltrating CD8^+^ T lymphocytes [84]. This was the first evidence in hTERT-based vaccination trials, that the induction of tumor-infiltrating T cells directly correlates with a clinical response.

In another study, Aloysius et al. [85] tested a vaccination protocol based on autologous DC pulsed with MHC-I-restricted p540 or p865 peptides with or without MHC class II cognate help (p766 and p672 hTERT peptides) and supplemented with TNF-α (with or without IFN-α) in different advanced cancer patients. The authors observed that class II cognate help significantly enhanced peptide-specific CD8^+^ T cell responses compared with class I pulsed DCs alone, whereas addition of IFN-α to ex vivo monocyte-derived DCs, did not significantly improve T cell responses, compared with TNF-α alone. [85]. This study further underlined the importance of T helper cells contribution in view of designing hTERT-based vaccines.

In a case report, Suso and co-workers treated a pancreatic adenocarcinoma patient with autologous DCs loaded with hTERT mRNA for three years, after radical surgery and chemotherapy with gemcitabine. The patient achieved a complete clinical remission and developed an immune response against several hTERT-derived Th and CTL epitopes. These results suggest the use of vaccination with DCs loaded with mRNA encoding a given antigen for the identification of clinically relevant immunogenic T cell epitopes [86].

In analogy to hTERT, survivin is a TAA implicated in cell cycle progression and apoptosis inhibition and is widely overexpressed in cancer cells, therefore, it is considered a promising target for anti-cancer immunotherapy. Berntsen and colleagues vaccinated 27 patients with metastatic renal cell carcinoma with DCs loaded with either a mix of survivin and hTERT peptides or tumor lysate in addition to low-dose IL-2. The vaccine was administered intranodally or intradermally. Although none of the patients developed an objective response, 13/27 patients obtained disease stabilization (5/13 HLA-A2-positive patients and 8/14 HLA-A2-negative patients). Apparently, disease stabilization was more frequent in patients vaccinated intradermally (SD in 7/9) than in patients vaccinated intranodally (SD in 6/18). An antigen-specific immune response was demonstrated in all HLA-A2-positive vaccinated patients and immune responses against telomerase peptides were stronger compared with survivin peptides [87].

In a phase II trial, Ellebaek and colleagues treated 28 metastatic melanoma patients with autologous DCs pulsed with survivin, hTERT and p53-derived peptides (HLA-A2-positive subjects) or tumor lysate (HLA-A2-negative patients). Concomitantly the patients were treated with IL-2, metronomic cyclophosphamide and celecoxib with the intent to improve antitumor immune response. Sixteen patients (57%) achieved disease stabilization, whereas induction of an antigen-specific immune response was observed in 9 out of 15 screened patients (HLA-A2-positive patients arm). Despite the use of metronomic cyclophosphamide, Treg cells did not decrease during treatment, but the number of patients obtaining clinical benefits (more than doubled SD and 6-month survival) significantly increased compared to a previous trial [88] using the same DC vaccine but without cyclophosphamide and celecoxib [89]. These data indicate that combining immunotherapy with treatments down-modulating tumor-induced immunosuppression may enhance the efficacy of such immunotherapies.

In a recent phase I clinical trial, DCs were pulsed with three distinct HLA-A2-restricted tumor peptides (hTERT, carcinoembryonic antigen and survivin) and administered to 12 patients with advanced pancreatic cancer. Peptide-loaded DCs vaccination was administered in combination with a TLR3 agonist (poly-IC:LC) as an adjuvant. Four out of the eight evaluable patients achieved disease stabilization, and antigen-specific immune response was observed in three patients with stable disease [90].

In a phase I/II trial on glioblastoma, DCs transfected with RNA purified from autologous cancer stem cell cultures in combination with hTERT and survivin mRNA were administered after the completion of standard post-operative chemo-radiotherapy. All treated subjects developed an immune response without significant toxicity or signs of autoimmunity. Vaccinated patients had significantly longer PFS compared to the historical-matched controls (694 days versus 236 days, *p* = 0.0018) and 5/7 patients were alive after two years follow-up [91].

Indoleamine 2,3-dioxygenase (IDO) an enzyme expressed by tolerogenic DCs plays an important role in immune escape mechanisms. Indeed, IDO activity, leading to kynurenine production through tryptophan degradation, suppresses effector T cell function and promotes the differentiation of Treg [92]. In a single patient report, a subject with metastatic melanoma pretreated with ipilimumab, an anti-CTLA-4 checkpoint inhibitor, was vaccinated with IDO-silenced DCs transfected with mRNA for survivin or hTERT tumor antigens. The authors observed T cell responses to survivin and hTERT, but also against other TAA (MART-1- and NY-ESO-1) supporting vaccine-induced epitope spreading [93].

GRNVAC1 (also known as AST-VAC1) is a DC-based vaccine developed for cancer immunotherapy. This vaccine relies on the administration of mature autologous DC transfected with mRNA encoding a chimeric lysosome-associated membrane protein-1 (LAMP)-hTERT protein. LAMP directs hTERT Ag processing into the MHC-II pathway, thus improving the activation of Ag-specific CD4^+^ T cells without affecting the intracellular generation and subsequent presentation of MHC class I epitopes [36]. Since CD4^+^ T cells play a crucial role in antitumor immune response, also this approach displays potential advantages in comparison to vaccination protocols that primarily activate CD8^+^ T cells. In vivo data further confirmed that patients with metastatic prostate cancer who received DC transfected with mRNA encoding the chimeric LAMP-hTERT protein developed significantly higher CD4^+^ CTL response as compared to patients who received DC transfected with the unmodified hTERT vaccine [94].

In acute myeloid leukemia patients (AML), administration of GRNVAC1 has been associated with hTERT specific immune response and improved disease-free survival, especially in high-risk patients, without major toxicities [95,96]. A long-term follow-up study on the same cohort of patients demonstrated that, after a median of 52 months, 58% of patients in complete remission (11 of 19 patients) were free of disease recurrence and 57% of the patients aged ≥ 60 years (4 of 7 patients) were free of disease recurrence at a median follow-up of 54 months [97].

GRNVAC2 is another cell-based vaccine in which DCs are obtained from human embryonic stem cells instead of from leukapheresis procedures, but to date, no published data are available on this approach.

## 4. Other Vaccination Approaches Targeting hTERT

Besides DCs, also B lymphocytes have been employed as APC for hTERT-targeted vaccination through a strategy termed “transgenic lymphocyte immunization”. In a phase I trial conducted at University of California San Diego, 15 prostate cancer patients were vaccinated with autologous transgenic B lymphocytes with pDNA encoding two hTERT peptides (p540 and p572Y), and an antigen-specific immunological response was observed in 80% of the patients [98,99].

At a pre-clinical level, other cell-based approaches targeting hTERT have been studied. These strategies include: DC transfected with adenovirus containing hTERT cDNA sequence, hTERT-expressing human umbilical vein endothelial cells (HUVECs), fibroblast-derived AAPCs (artificial antigen presenting cells) transduced with hTERT-encoding cDNA, as well as vaccination with mannan-modified adenovirus encoding hTERT targeting DCs in vivo. All these vaccination models showed encouraging results concerning their ability to induce robust antigen-specific immune responses [37,100,101,102,103].

Among different techniques applied to induce immunization against hTERT, DNA-based vaccines have also been explored and are currently under investigation. phTERT is a synthetic, full-length hTERT DNA vaccine delivered by electroporation, that has been tested in mice and non-human primates demonstrating to be cable of eliciting potent cytotoxic responses [104]. A Phase I study (NCT02960594) is currently ongoing in patients with a different solid tumor cancer to investigate the safety and immunogenicity of an hTERT-encoding DNA vaccine (INO-1400/INO-1401) alone or in combination with INO-9012 (a plasmid encoding for IL-12) delivered by electroporation [68]. A two phase I/IIA trial is currently testing INO-5401 (a mixture of three synthetic plasmids targeting Wilms’ tumor gene-1, prostate-specific membrane antigen and hTERT) and INO-9012 in combination with atezolizumab (anti-PD-L1) in advanced urothelial carcinoma (NCT03502785) or in association with cemiplimab (anti-PD-1) and chemoradiotherapy in glioblastoma (NCT03491683) [68]. Interestingly, a phase Ib study is testing INO-5401 alone or combination with INO-9012 as a preventive vaccination for women bearing mutations in BRCA1 or BRCA2 genes [68]. This latter example extends the role of hTERT-based vaccination as an immunoprevention tool applicable to healthy individuals at high cancer risk, due to genetic factors or medical history. INVAC-1 is an optimized DNA plasmid encoding an inactivated form of hTERT that is administered intradermally by gene electrotransfer. INVAC-1 has been tested in a variety of mouse models where it elicited antigen-specific cellular immune responses, including T helper and cytotoxic T cells, capable of slowing tumor growth and increases the survival rate of tumor-bearing mice [105,106]. Teixeira et al. designed the first-in-human Phase I study by using INVAC-1 as the only therapeutic agent in different advanced solid tumors. The authors report that treatment was well tolerated, disease stabilization was observed in 58% of the patients, and anti-hTERT specific CD8^+^ and CD4^+^ T cell responses were detected in 25% and 63% of the patients, respectively [107]. A phase II study (NCT03265717) exploring the efficacy of INVAC-1, either as a single agent or in combination with ibrutinib (an inhibitor of Bruton’s tyrosine kinase), in patients with Chronic Lymphocytic Leukemia is currently ongoing [68].

Gene-modified T cell therapy has been developed to optimize the delivery of effector cells targeting tumor antigens displayed on the surface of cancer cells. This approach is based on the adoptive transfer of T cells that are genetically manipulated in order to express TCRs with high affinity for a given tumor antigen. TCR-engineered T cells designed to specifically recognize telomerase have been explored, but current data, although encouraging, are limited to pre-clinical experimental settings [108,109,110,111,112].

## 5. Discussion

Data derived from the huge amount of pre-clinical experiments demonstrated that: (i) Telomerase is expressed in the large majority of human cancers at every phase of tumor progression, whereas it is virtually non-detectable in normal adult somatic cells; (ii) hTERT-derived peptides are expressed by cancer cells in the context of MHC-I and these peptide-MHC complex are able to stimulate CD8^+^ CTLs; (iii) MHC-II-binding TERT peptides can be presented by APCs and mediate the activation of CD4^+^ T helper cells; (iv) hTERT-specific T cell precursors can be isolated from the blood of healthy subjects and cancer patients and display tumoricidal activity against hTERT-expressing cancer cell lines and primary cancer cells; (v) hTERT-specific T cell precursors can be efficiently and safely expanded in animal models through different vaccination strategies. All these encouraging premises paved the way for the clinical development of different hTERT vaccination strategies.

To date, data from 33 clinical trial exploring safety and efficacy of immunotherapies targeting telomerase are available in the literature (26 Phase I–I/II, 6 phase II, and 1 phase III trials). Phase I studies are intended to evaluate toxicity and determine the optimal dose and schedule of the investigational drug, as well as to reveal preliminary antitumor activity. Phase II trials mainly estimate anti-tumor efficacy of a given treatment, further defining its safety. Phase III studies are randomized trials designed to determine whether a new drug provides clinical benefit compared to the current standard of care [114]. Clinical studies on hTERT are based on different vaccination approaches and have been applied in solid tumors and hematologic malignancies of different histologies. The majority of the trials are based on the administration of single or multiple hTERT synthetic peptides restricted to MHC-I (e.g., p540) and/or MHC-II molecules (e.g., GV1001) in order to induce CTL (CD8^+^) or T helper (CD4^+^) immune responses, respectively. In a minority of the studies, APC, generally DCs, pulsed with hTERT peptide(s) or transfected with hTERT encoding mRNA/DNA have been employed. In some studies, hTERT-based immunotherapy has been administered in association with standard anti-cancer treatments, such as chemotherapy and/or radiotherapy, or in combination with drugs aimed at counteracting tumor-related immunosuppressive pathways. hTERT-specific immune responses are reported in the majority of the studies in high percentages of the vaccinated subjects. Nevertheless, objective clinical responses or, at least, disease stabilization have been successfully achieved only in a minority of the studies in a fluctuating proportion of the treated subjects (ORR in 8–77% and SD in 16–83% of the patients). Collectively, data from clinical trials indicate that hTERT-based immunotherapies can efficiently induce specific T cell responses in the majority of the patients, but on the whole, clinical results are generally modest.

Optimization strategies potentially capable of improving the efficacy of hTERT-targeting immunotherapy may be addressed to vaccine formulation, patients’ selection, qualitative characteristics of the immune response and immunosuppressive barriers negatively influencing anti-tumor T cell responses (Table 2). To improve their immunogenicity, vaccines should include multiple immunogenic MHC-I and MHC-II-restricted hTERT epitopes in order to expand CD8^+^ and CD4^+^ TERT-specific T cell precursors. Indeed, CD4^+^ T helper cells are crucial to elicit a strong and durable immune response against cancer cells. In addition, multi-epitope cancer vaccines, expanding HLA alleles coverage, increase the number of patients who may benefit from given immunotherapy. Many tumor-associated antigens, including telomerase, are self-antigens since they are also expressed by normal tissues. As a consequence, T cells capable of recognizing these antigens with high-avidity are very rare, since they are normally eliminated through thymic deletion. In this context, optimized cryptic peptides represent an attractive technology in order to increase the number of T cells triggered by immunotherapy (e.g., Vx-001 vaccine). In vaccinology, adjuvants enhance the immune response to the vaccine antigen(s) by activating innate immune cells (e.g., DCs), a crucial step for effective T cells priming. Therefore, selection of the adjuvant should be considered as important as the choice of the immunizing antigen(s), and the improvement of adjuvant efficiency is critical for inducing a protective and long-lasting immune response against weak immunogenic antigens, such as telomerase. Different studies correlated hTERT-specific T cell responses with clinical responses, in particular, immune responders generally display overall survivals significantly increased as compared to non-responders. This observation suggests that pre-selecting patients able to mount an adequate immune response upon vaccination may increase clinical benefits from hTERT-based immunotherapies. For example, selection criteria should take into account the presence of mutations in the hTERT-promoter region, the levels of hTERT antigen in cancer cells, the stage and the histology of the tumors to be treated. To eradicate cancer cells, effector immune cells must first be relieved from the multiple suppressive barriers of the tumor microenvironment (TME). TME consists of tumor cells, stromal cells, immune cells and extracellular matrix, and it is strictly implicated in tumor growth and progression, as well as in tumor response to different treatments. The non-malignant cells of the TME, such as regulatory lymphocytes and immunosuppressive myeloid cells, have a dynamic and tumor-promoting effect at all stages of carcinogenesis. As a consequence, even if immune effector cells are recruited to the tumor site, their anti-tumor functions are blunted by the immunosuppressive milieu of the TME. Targeted manipulation of the immunosuppressive network of the TME can be obtained through different pharmacological approaches, thus potentially improving the efficacy of anti-cancer immunotherapies. Finally, most T cells in TME are polarized into exhausted T cells, express high levels of inhibitory receptors and display decreased cytokine production and effector functions, thus losing the capacity to eliminate cancer cells. Interestingly, recent therapeutic strategies, such as inhibitory checkpoints blockade, demonstrated to be effective in reactivating these exhausted T cells. A combined therapeutic protocol, including hTERT immunotherapy with immune-checkpoint inhibitors, could potentially increase the effectiveness of the vaccination and is currently under investigation in different clinical trials.

## 6. Conclusions

Collectively, data obtained from over fifteen years of hTERT-vaccine clinical trials indicate that these immunotherapies may represent a promising approach in cancer treatment. However, new strategies are required to improve the clinical efficacy of telomerase vaccination. Knowledge on anti-tumor immunity has advanced rapidly in recent years, and combining vaccination with targeted manipulation of the immune response through different pharmacological approaches will be able to improve the efficacy of hTERT-based anti-cancer immunotherapies in future trials.

## Figures and Tables

**Table 1:** Clinical trials on human telomerase reverse transcriptase (hTERT)-targeting immunotherapies.

Study Design (NCT When Available)	Target	Vaccine Approach	Additional Treatments	Outcomes	Year	Ref(s)
**Peptide vaccines**						
Phase I	Different metastatic solid tumors	p540 + Montanide		IR: 7/13 patients; no OR, Aes: well-tolerated	2004	[48]
phase I	Pancreatic cancer	GV1001 + Imiquimod		IR: 6/13 patients; Aes: well-tolerated	2005	[113]
phase I/II trial (CTN-2000)	Non-small-cell lung cancer	GV1001 + p540 + GM-CSF		IR: 13/24 subjects; 4/24 long-time survivors; 2/24 patients free of disease; Aes: well-tolerated	2006	[49,55]
phase I/II	Pancreatic cancer	GV1001 + GM-CSF		IR: 24/38 patients; Aes: well-tolerated	2006	[54]
Phase I	Various advanced solid tumors	Vx-001		IR: 13/14 patients; SD in 4/19 patients; Aes: well-tolerated	2006	[75]
Phase I	Breast cancer	p540 + GM-CSF		IR: 9/16 patients (increased median OS); hTERT-specific TIL in 3/6 patients; no OR; SD in 9/18 patients; Aes: well-tolerated	2007	[46]
Phase I/II	Non–small-cell lung Cancer	Vx-001		IR: 16/21 patients after two vaccinations and 10/11 patients after six vaccinations; SD in 8/22 patients; Aes: well-tolerated	2007	[76]
phase II NCT00444782	Hepatocellular carcinoma	GV1001 + GM-CSF	Cyclophosphamide	IR: 0/40, 0/40 complete or partial response; SD in 17/37 of the patients; Aes: well-tolerated	2010	[60]
phase I/II	cutaneous T cell lymphoma	GV1001 + GM-CSF		IR: 1/6; 0/6 OR (1/6 disease progression)	2011	[59]
Phase I	Melanoma	Group A: GV1001 + p540 + GM-CSF *versus* Group B: GV1001 + PPD23		IR: 7/10 in Group A *versus* 0/6 in Group B; Aes: well-tolerated	2011	[56]
phase I/II	Melanoma	TERT p611 (GV1001)	Temozolomide	IR: 18/23 evaluated subjects, 5/25 partial tumor regression, SD in 6/25 subjects, CR in 1/25 patients, Aes: well-tolerated	2011	[57]
Phase II trial (CTN-2006)	Non-small-cell lung cancer	GV1001 + TERT p540 + GM-CSF	Vaccination after radiotherapy + docetaxel	IR: 16/20 patients, Aes: well-tolerated	2011	[55]
Phase I	Various advanced solid tumors	two injections of p572Y followed by four injections of 572 (scheme A) or 572Y (scheme B)		IR: higher T cell responses (44% vs. 17%) and number of specific T cells in group A	2011	[74]
Phase I/II NCT00499577	Multiple myeloma	p540, p572Y and p988Y + survivin + Montanide + GM-CSF (only HLA-A2+ patients)	All patients received PCV, ASCT and autologous activated T cells	IR: 10/28 patients (hTERT vaccine arm); no differences in EFS and OS between the two groups; Aes: well-tolerated	2011	[81]
phase I/II	Various advanced solid tumors	Vx-001		IR: 27/53 patients after two vaccinations and 10/11 patients after six vaccinations; Aes: well-tolerated	2012	[77]
Phase II	Various advanced solid tumors	Vx-001		IR: 30/55 patients after two vaccinations and 24/36 patients after six vaccinations; SD in 18/55 patients; OR in 2/55 patients (1 CR and 1 PR); Aes: well-tolerated	2012	[78]
Phase I	Prostate or renal cancers	p540, p672, p766 and p611 + Montanide + Imiquimod		IR: 11/11; SD in 4/11; increased OS in immune-responders; Aes: well-tolerated	2013	[67]
phase I/II	Pancreatic cancer	GV1001 + GM-CSF + gemcitabine concurrently (groups A/B) or added at progression (group C)		IR group A/B: 8/12, group C: 2/5; stable disease group A/B: 10/12, group C: 1/5; 0/17 CR or PR, Aes: well-tolerated	2014	[58]
Phase III	Pancreatic cancer	GV1001 + GM-CSF	Gemcitabine + Capecitabine (alone, with sequential GV1001 or with concurrent GV1001)	IR: 12/32 patients on sequential immunotherapy and 25/68 patients on concurrent chemoimmunotherapy; no differences in OS, median time to progression or OR among the three groups; Aes: well-tolerated	2014	[62]
Phase II	Non–small-cell lung Cancer	Vx-001		IR 23/35 patients; SD in 13/46 patients; PR in 3/46 patients; Aes: well-tolerated	2014	[79]
Phase I	Hepatocellular carcinoma	p461 + Montanide		IR: 10/14 patients (effector-memory phenotype); no HCC recurrence in 5/11 responding patients; Aes: well-tolerated	2015	[47]
Phase I/II NCT01784913	Prostate cancers	UV1 + GM-CSF	Radiotherapy + androgen deprivation	IR: 18/21 evaluable patients; SD in 16/21 patients; OR in 10/21 patients; Aes: generally well-tolerated	2017	[69]
Phase Iib NCT01935154	Non–small-cell lung Cancer	Vx-001 *versus* placebo	Maintenance immunotherapy after chemotherapy	IR: 22/75 patients; no CR or PR; no OS increase in Vx-001 groups; increased OS and TTF in immune-responders; Aes: well-tolerated	2020	[80]
**Dendritic cell vaccines**						
Phase I	Advanced breast and prostate carcinoma	DC pulsed with p540 hTERT and KLH		IR: in 4/7 patients; one partial regression	2004	[84]
Phase I	Prostate cancer	B lymphocytes transfected with pDNA encoding hTERT peptides (p540 and p572Y)		IR: 12/15 patients	2004	[98,99]
Phase I	Prostate cancer	GRNVAC1	DCs transfected with LAMP + hTERT mRNA	IR: 19/20 subjects; Aes: well-tolerated	2005	[94]
Phase I/II	Renal cell carcinoma	DCs pulsed with hTERT and survivin peptides (HLA-A2+ patients) or tumor lysate (HLA-A2- patients) + low-dose IL-2		IR: 6/6 subjects (HLA-A2+ patients); SD in 13/27 patients; Aes: well-tolerated	2008	[87]
Phase I	Various advanced solid tumors	DC pulsed with p540 or p865 ± MHC-II-restricted peptides (p766 and p672)		IR: class II cognate help increased peptide-specific CD8^+^ T cell responses; partial clinical responses in 4/16 patients; Aes: well-tolerated	2009	[85]
Phase II NCT00510133	Acute Myeloid Leukemia	GRNVAC1	DCs transfected with LAMP + hTERT mRNA	IR: 11/19 subjects; favorable disease-free survival (long term follow-up); Aes: well-tolerated	2009	[95,96,97]
Phase I/II	Melanoma	DCs pulsed with survivin + hTERT + p53 peptides (HLA-A2+ patients) or tumor lysate (HLA-A2- patients) + IL-2 + IFN-α		IR: 6/10 HLA-A2+ patients; SD in 11/46 evaluable patients; Aes: well-tolerated	2010	[88]
Phase II NCT00197912	Melanoma	DCs pulsed with survivin + hTERT + p53 peptides (HLA-A2+ patients) or tumor lysate (HLA-A2- patients) + IL-2	Metronomic cyclophosphamide and celecoxib	IR: 9/15 HLA-A2+ patients; SD in 16/28 patients; Aes: well-tolerated	2012	[89]
Phase I/II NCT00846456	Glioblastoma	DCs transfected with mRNAs from CSCs lysates + hTERT and survivin mRNA	Post-operative chemo-radiotherapy (EORTC regimen)	IR: 7/7 patients; longer PFS, no difference in OS or tumor volume; Aes: well-tolerated	2013	[91]
Phase I NCT01410968	Pancreatic adenocarcinoma	DCs pulsed with three tumor peptides (hTERT, CEA and survivin) + poly-IC:LC		IR: 3/8 patients; SD in 4/8 subjects; Aes: well-tolerated	2017	[90]
**DNA vaccines**						
Phase I NCT02301754	Various advanced solid tumors	INVAC-1		IR: CD8 and CD4 T cell responses in 25% and 63% of patients, respectively; SD in 15/26; no CR or PR; Aes: well-tolerated	2020	[107]

Aes, adverse events; ASCT, autologous stem cell transplantation; CEA, carcinoembryonic antigen; CR, complete response; CSCs, cancer stem cells; DC, Dendritic Cell; EFS, event-free survival EORTC, European Organization for Research and Treatment of Cancer; GM-CSF, granulocyte-macrophage colony-stimulating factor; hTERT, human telomerase reverse transcriptase; HLA, human leukocyte antigen; IL, interleukin; IR, immunological response; KLH, keyhole limpet hemocyanin; LAMP, lysosome-associated membrane glycoprotein; MHC, major histocompatibility complex; PFS, progression-free survival; OR, objective responses; OS, overall survival; PCV, pneumococcal conjugate vaccine; PR, partial response; SD, stable disease; TTF, time to treatment failure.

**Table 2:** Strategies to increase the efficacy of hTERT-based immunotherapies.

Target	Intervention(s)	Objective(s)	Example(s)	Ref(s)
Vaccine formulation	Multiple immunogenic MHC-I and MHC-II-restricted hTERT epitopes	Expand both CD8^+^ and CD4^+^ TERT-specific T cells Extended HLA-haplotype coverage	DNA or RNA vaccines, multi-peptide vaccines (e.g., GX301)	[44,45,66,67,115,116]
	Optimized cryptic peptides	Escape self-immune tolerance (since they are cryptic) and are immunogenic (via structural modification increasing their affinity for MHC)	Vx-001	[71,74]
	Adjuvant(s)	Adjuvants enhance the immune response to antigen by activating innate immune cells	GM-CSF, IL-12, TLR-agonists (e.g., Imiquimod, poly-IC:LC) Use of multiple molecules with complementary actions	[67,90,116,117,118]
Selection of patient candidates for immunotherapy	Select patients with TERT-promoter mutations or high TERT-antigen expression	Increase cancer cells susceptibility to hTERT-specific T cells		[115,119]
	Patients with early-stage tumors	Vaccinations are less effective in advanced tumors in “immune escape” phase		[115]
Immune responses induced by vaccination	Optimizing antigen dose Effective T cell co-stimulation (e.g., 4-1BB, OX40, CD40, CD27) Cytokines (e.g., IFNs, IL-7, IL-15) and immunomodulatory molecules (e.g., mTOR inhibitors)	Improve functional characteristics of the induced lymphocytes (e.g., memory T cells)		[115,116,120,121]
Tumor microenvironment	Treg cells depletion (e.g., anti-CTLA-4, anti-OX40, low continuous metronomic cyclophosphamide, CCR4 antagonists) Myeloid-derived suppressor cells (e.g., TRAIL receptor agonists, PDE5-i, NO-releasing aspirin, ATRA, sunitinib) Inhibition of metabolic targets (e.g., IDO, adenosine axis, arginase, nitric oxide synthase) Vessel normalization and hypoxia reduction (e.g., anti-angiogenetic drugs, HIF inhibitors) T cells resuscitation via checkpoints blockade (anti-CTLA-4, -PD-1 and -PD-L1 mAbs alone or in combination) Radiation therapy and/or chemotherapy (intended as TME modulators)	Counterbalancing multiple suppressive barriers	GV1001 + GM-CSF + gemcitabine, tadalafil + radiation therapy in pancreatic cancer (NCT01342224) UV1 + anti-CTLA-4 (ipilimumab) in melanoma (NCT03538314) UV1 + anti-PD-1 (pembrolizumab) in melanoma (NCT02275416) UV1 + anti-CTLA-4 (ipilimumab) + anti-PD-L1 (nivolumab) in mesothelioma (NCT04300244) or in melanoma (NCT04382664)	[122,123,124,125,126,127,128]

ATRA, all-trans retinoic acid; CTLA-4, cytotoxic T-lymphocyte-associated antigen 4; HIF, hypoxia-inducible factors; GM-CSF, granulocyte-macrophage colony-stimulating factor; hTERT, human telomerase reverse transcriptase; IL, interleukin; IDO, indoleamine 2,3-dioxygenase; mAbs, monoclonal antibodies; MHC, major histocompatibility complex; NO, nitric oxide; PD-1, programmed cell-death protein 1; PD-L1, programmed death-ligand 1; PDE5i, phosphodiesterase type 5 inhibitors; TLR, toll-like receptor; TRAIL, tumor necrosis factor-related apoptosis-inducing ligand. NCT numbers can be entered into the search box at http://clinicaltrials.gov/ for a detailed description of the study.
